# Supplementary material for: Potential Biomarkers and Endometrial Immune Microenvironment in Recurrent Implantation Failure
Source: Biomolecules. 2023 Feb 21;13(3):406. doi: 10.3390/biom13030406 (PMC10046278; doi:10.3390/biom13030406)
Supplement: Supplementary file 1 [file biomolecules-13-00406-s001.zip › Supplementary Table S1-S3; Figure S1,S5-S6.pdf]

Supplementary tables

Table S1. Baseline characteristics and inclusion and exclusion criteria for RIF patients and fertile controls in the GSE111974 dataset.

Table S2. Baseline characteristics and inclusion and exclusion criteria for RIF patients and fertile controls in the GSE92324 dataset.

Table S3. Gene Primers for RT-qPCR.

Table S1. Baseline characteristics and inclusion and exclusion criteria for RIF patients and fertile controls in the GSE111974 dataset [1].

	**Control (n = 24)**	**RIF (n =24)**	***p* value**
Age (years)	31.13 ± 3.86	32.76 ± 2.33	NS
BMI (kg/m^2)	23.15 ± 1.65	25.36 ± 1.43	NS
LH + day	7–10	7–10	-
Day 3 FSH (mIU/mL)	7.55 ± 1.67	6.52 ± 3.18	NS
Day 3 E2 (mIU/mL)	61.86 ± 25.56	56.41 ± 65.10	NS
Day 3 prolactin (ng/mL)	14.69 ± 5.66	12.45 ± 4.17	NS
Day 3 TSH (µIU/mL)	2.16 ± 1.42	2.28 ± 1.04	NS
Inclusion criteria	having a history of ≥1 live birth	failure of pregnancy in ≥3 consecutive IVF cycles with ≥1 transfer(s) of good quality embryo in each cycle	-
Exclusion criteria	active pelvic infections, undiagnosed vaginal bleeding, uterine anomalies, endometriosis, karyotype anomalies, and under any medications		-

BMI, body mass index; LH, luteinizing hormone; FSH, follicle-stimulating hormone; E2, estradiol; TSH, thyroid stimulating hormone; RIF, recurrent implantation failure; NS, nonsignificant.

Table S2. Baseline characteristics and inclusion and exclusion criteria for RIF patients and fertile controls in the GSE92324 dataset [2].

	**Control (n = 8)**	**RIF (n =10)**
Age (years)	20–40	20–40
HCG + day	6–7	6–7
LH, FSH, E2, P4, AMH, and prolactin	normal	normal
Normal endometrial thickness	8 mm	8 mm
Inclusion criteria	having a history of full-term normal delivery	failure of pregnancy in ≥2 consecutive IVF cycles with ≥4 transfer(s) of good quality embryo
Exclusion criteria	polycystic ovarian disease, endometriosis, hormonal abnormalities, autoimmune disorders, and male infertility	

HCG, human chorionic gonadotropin; LH, luteinizing hormone; FSH, follicle-stimulating hormone; E2, estradiol; P4, Progesterone; AMH, anti-Mullerian hormone; RIF, recurrent implantation failure.

Table S3. Gene Primers for RT-qPCR.

Gene	Sequence (5' -> 3')
NRCAM (Human)	Forwards: CTTCCAAGGCAGCAAGACTC<br>Reverse: CTTCAAAGACGAGGGAGCAC
ATP13A4 (Human)	Forwards: GCTGCTCTACTGGGAGACAAA<br>Reverse: CACCAGCTTAGGGTAGGCAC
TRIB2 (Human)	Forwards: ATGAACATACACAGGTCTACCCC<br>Reverse: GGGCTGAAACTCTGGCTGG
SHROOM3 (Human)	Forwards: CCCTCTCGGGGCGTCTAGCC<br>Reverse: GCCCAGCACTACTCGCTCCC
FAM155B (Human)	Forwards: CACGAAAGGCTGTAAGGCTG<br>Reverse: TCGGGGAGTATAAAGGGGCA
ZNF436 (Human)	Forwards: TAAGCCCAGTAGTGTCAGTTT<br>Reverse: CATTATAGTACCCACCCAGAG
SLC25A29 (Human)	Forwards: GGGGAGGACGAGGAGGAAA<br>Reverse: GCCTGCCACACCCCC
PDZD8 (Human)	Forwards: AAGACCCGCTGATCGACTTC<br>Reverse: CTTGCAAGGTCTGGTATGGAAA
RAB32 (Human)	Forwards: TGGGACAGCAGGACTCTGG<br>Reverse: ACTCGGGTCATGTTGCCAA
HOXB3 (Human)	Forwards: ATGCAGGGCAGTCCGGTGTA<br>Reverse: GGTGATGGGAAAGGTGGTTG
ACTB (Human)	Forwards: GTGGCCGAGGACTTTGATTG<br>Reverse: CCTGTAACAACGAATCTCATATT

References

1. Bastu, E.; Demiral, I.; Gunel, T.; Ulgen, E.; Gumusoglu, E.; Hosseini, M.K.; Sezerman, U.; Buyru, F.; Yeh, J. Potential Marker Pathways in the Endometrium That May Cause Recurrent Implantation Failure. *Reprod Sci* **2019**, *26*, 879-890, doi:10.1177/1933719118792104.
2. Pathare, A.D.S.; Zaveri, K.; Hinduja, I. Downregulation of genes related to immune and inflammatory response in IVF implantation failure cases under controlled ovarian stimulation. *American Journal of Reproductive Immunology (New York, N.Y. : 1989)* **2017**, *78*, doi:10.1111/aji.12679.

Supplementary figures

Figure S1. Construction of co-expression network.

Figure S5. The intersection of the 85 DEGs and 238 ERA genes.

Figure S6. Heatmap of the correlations between immune cells in the GSE111974 dataset.

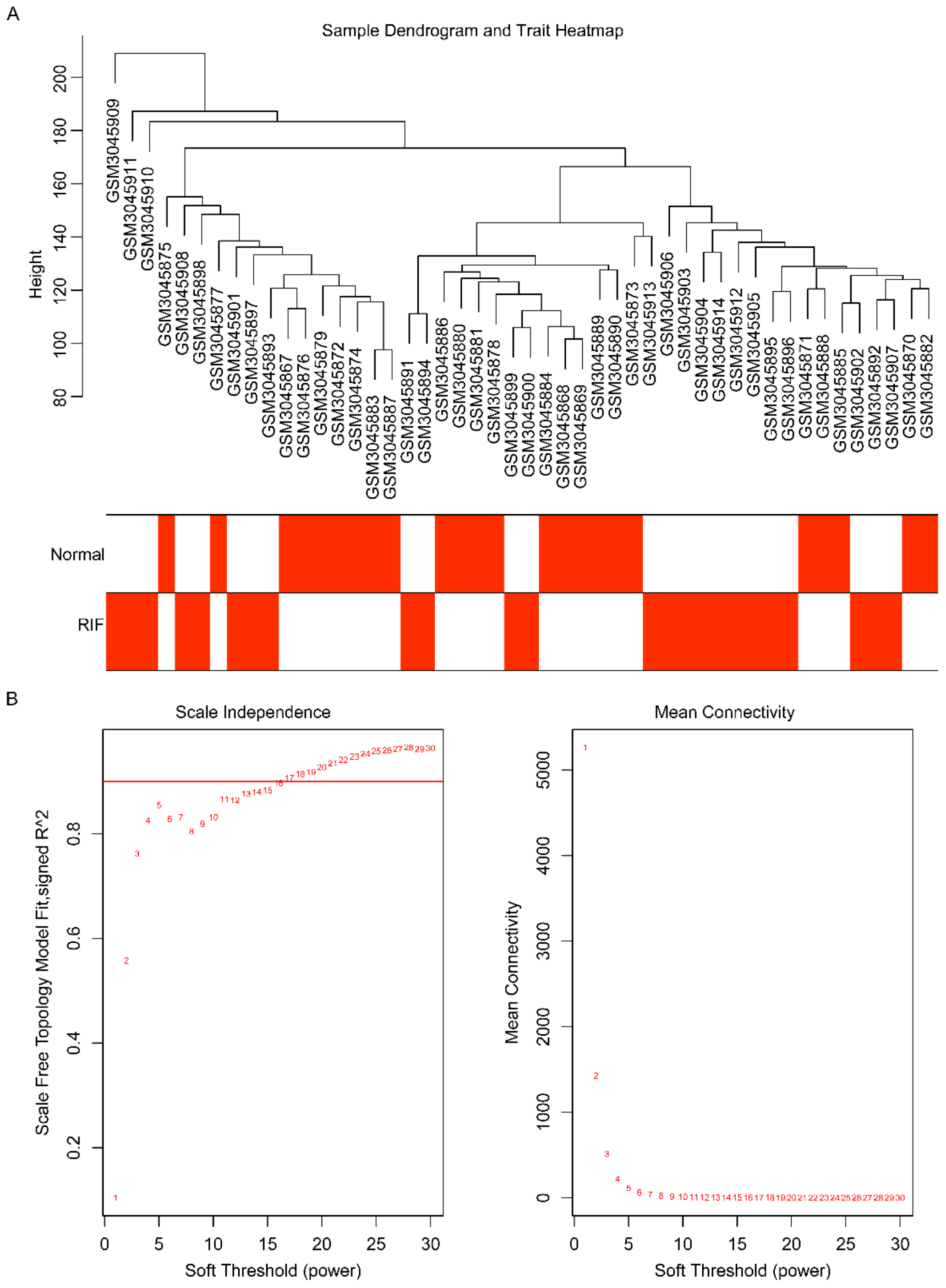

Figure S1. Construction of co-expression network. (A) Sample dendrogram and trait heatmap. (B) Analysis of the scale free topology model fit and the mean connectivity of different soft threshold powers.

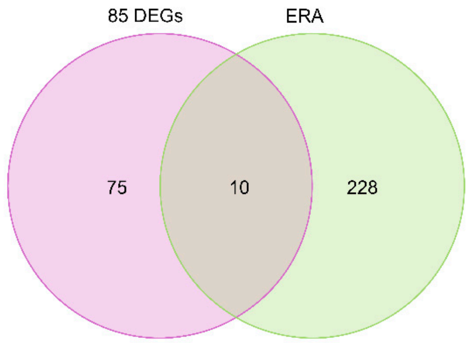

Figure S5. The intersection of the 85 DEGs and 238 ERA genes.

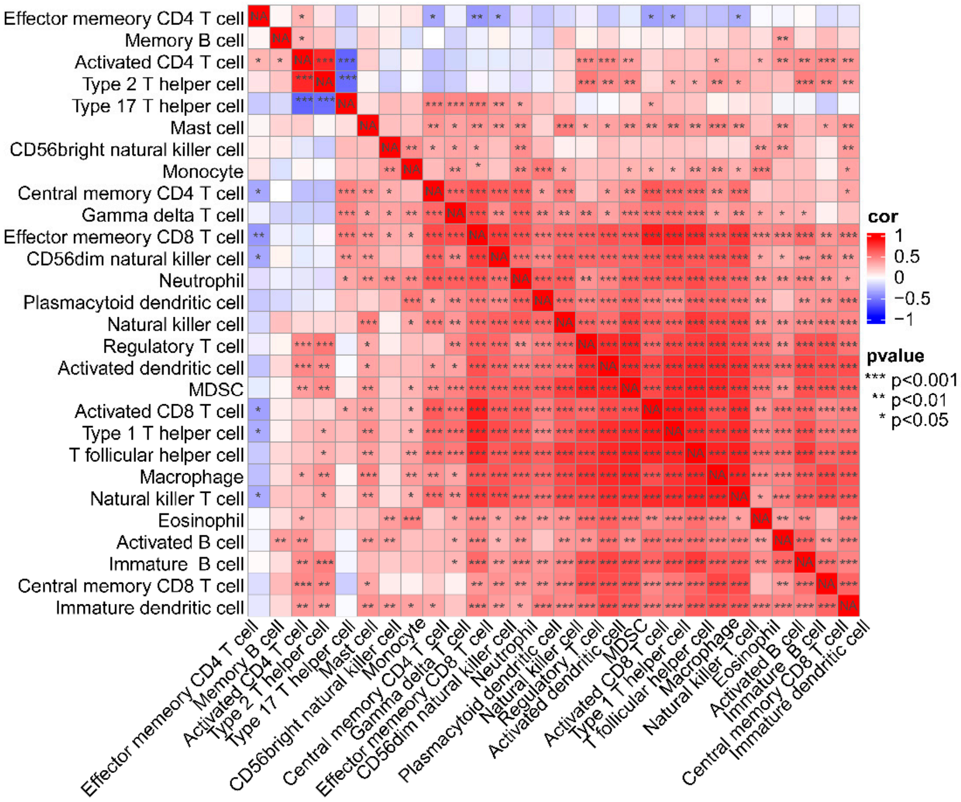

Figure S6. Heatmap of the correlations between immune cells in the GSE111974 dataset.
