# Supplementary figures and images for: Potential Biomarkers and Endometrial Immune Microenvironment in Recurrent Implantation Failure
Source: Biomolecules. 2023 Feb 21;13(3):406. doi: 10.3390/biom13030406 (PMC10046278; doi:10.3390/biom13030406)

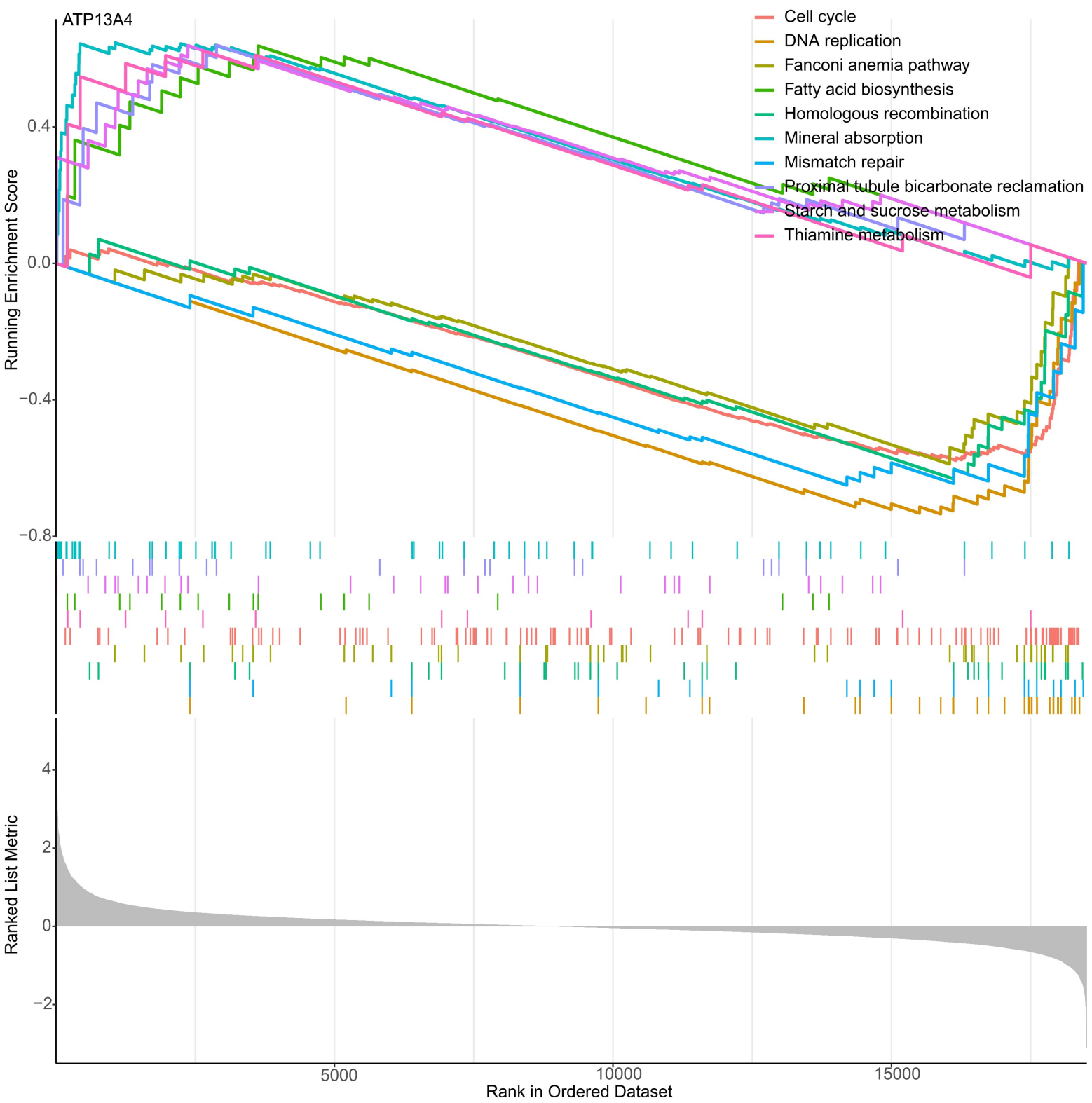

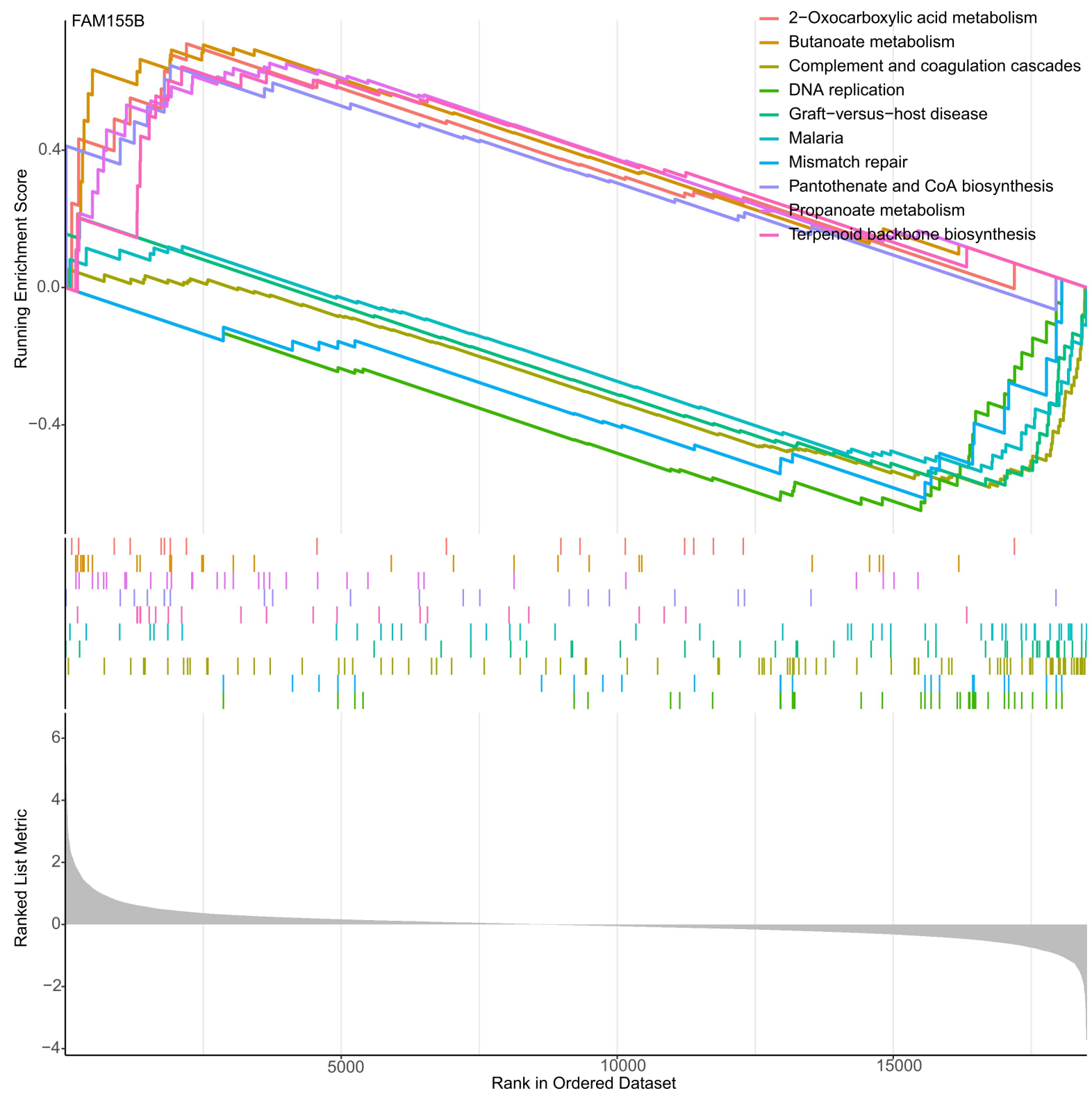

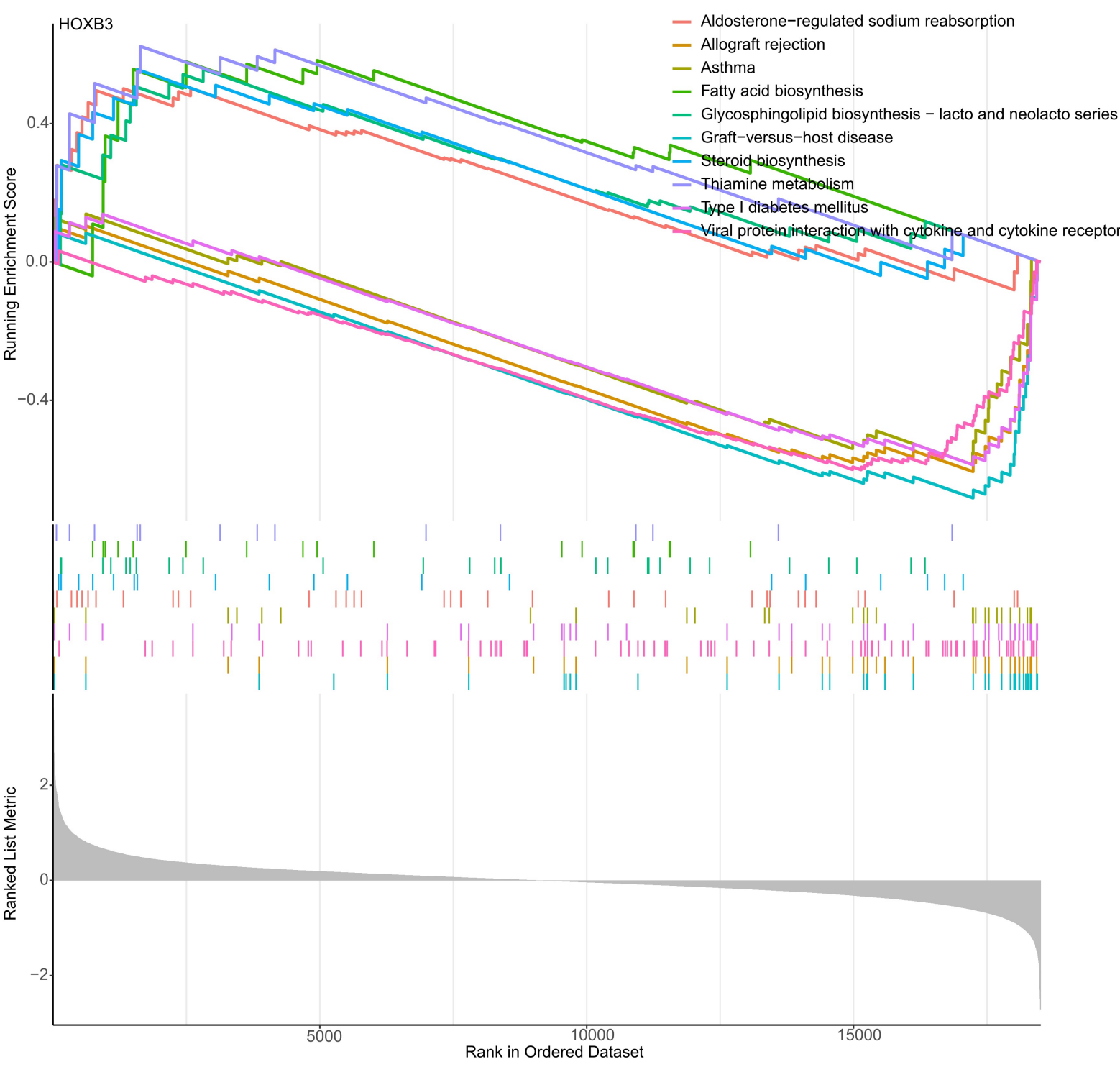

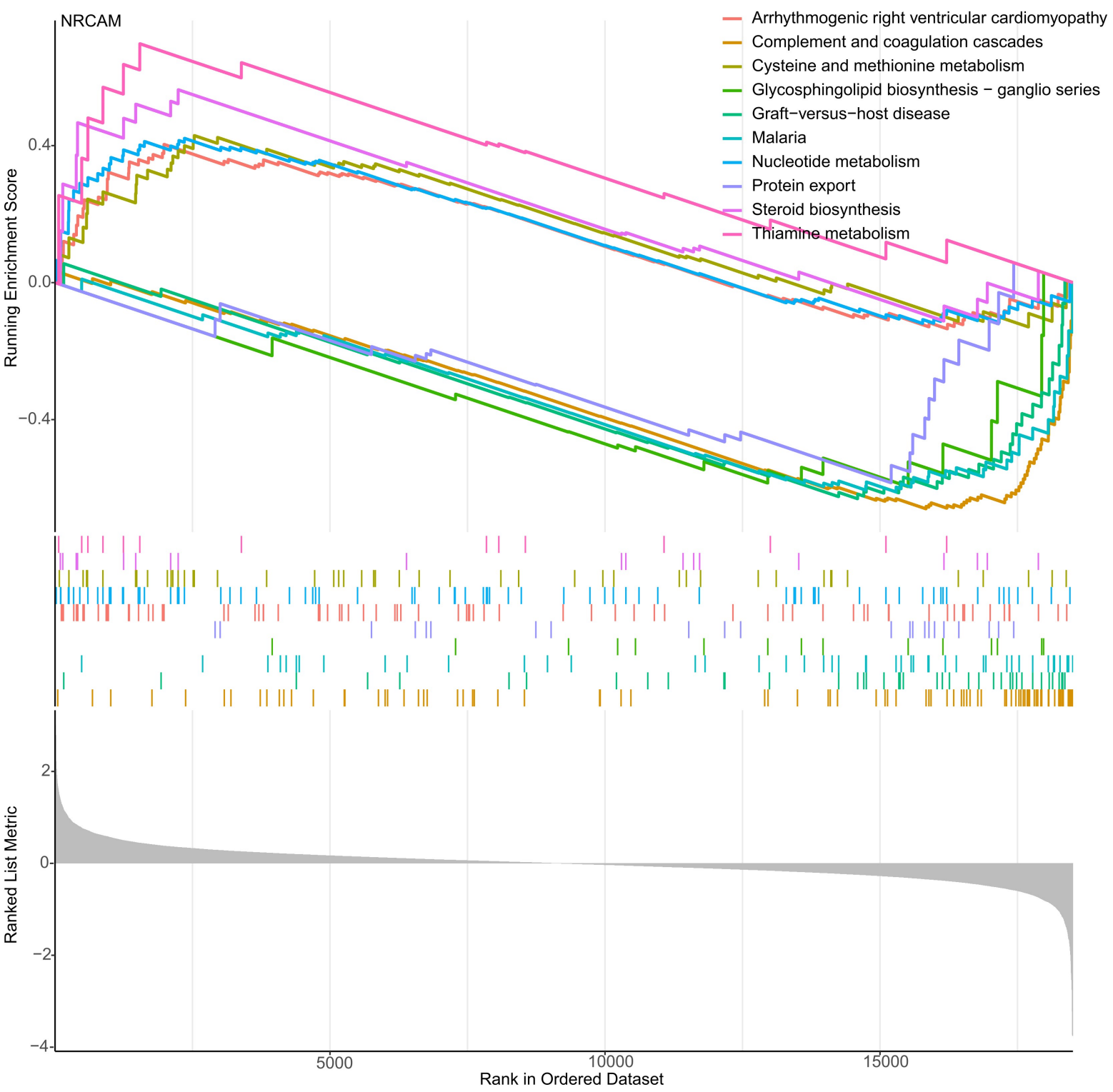

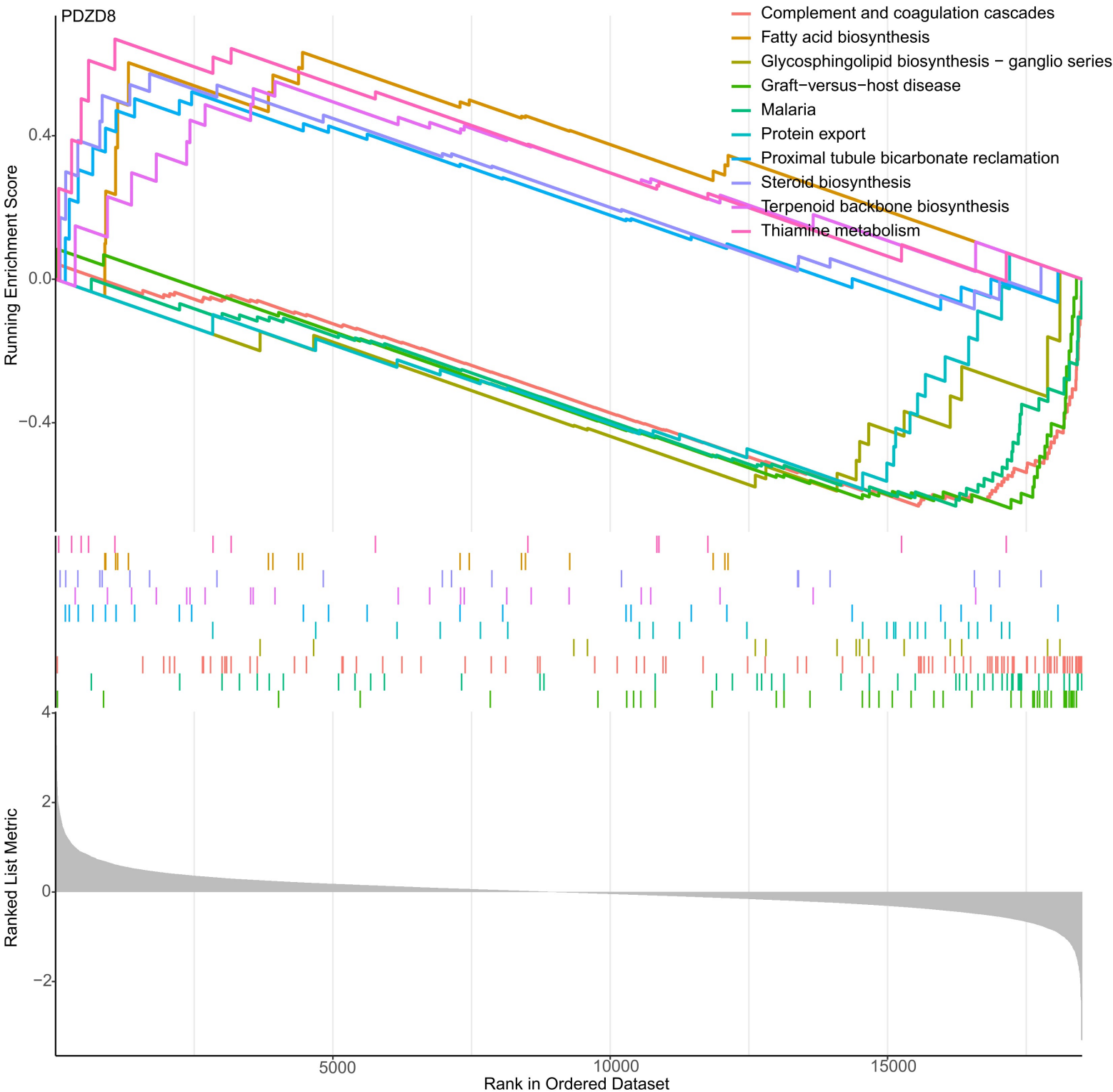

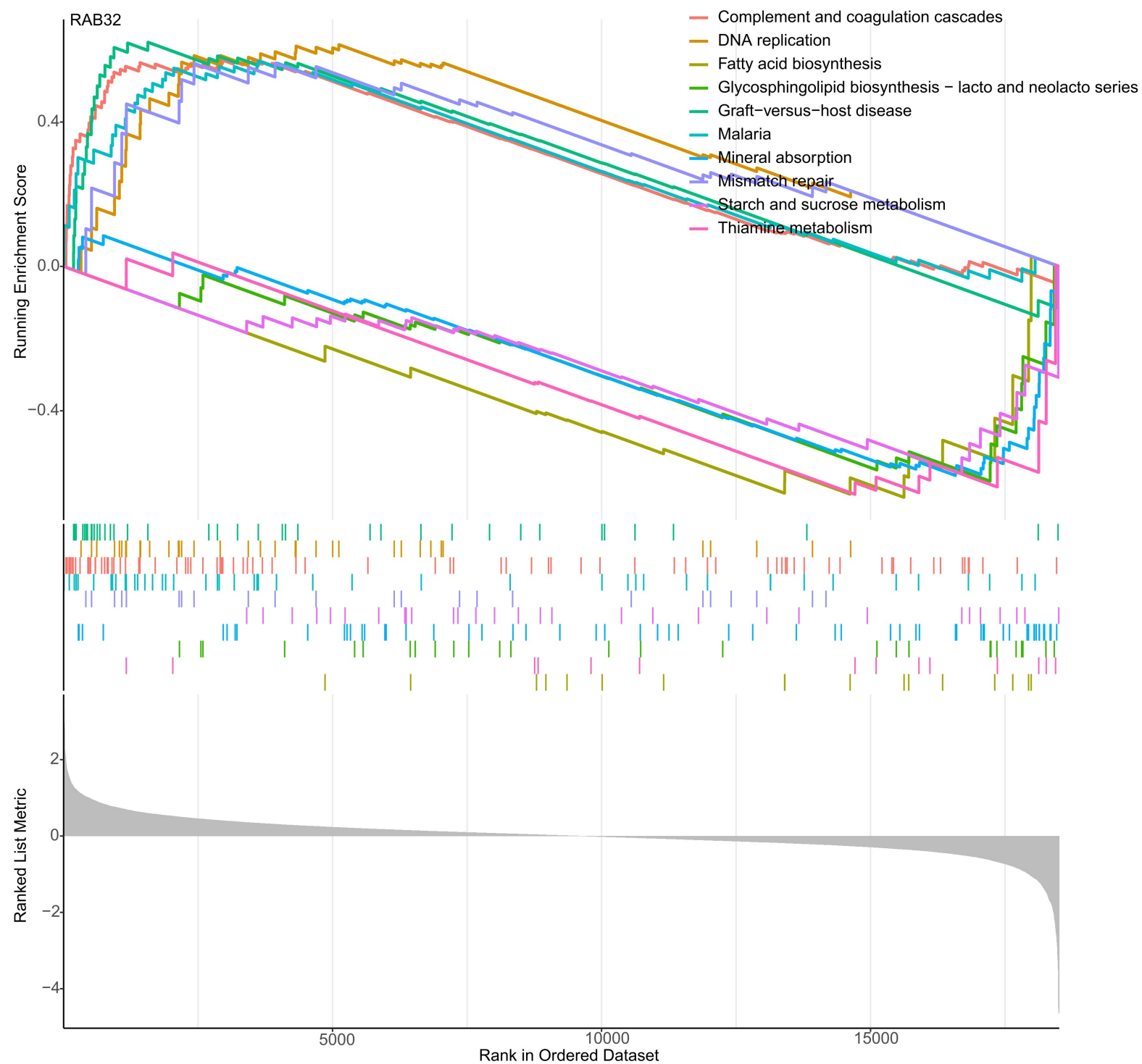

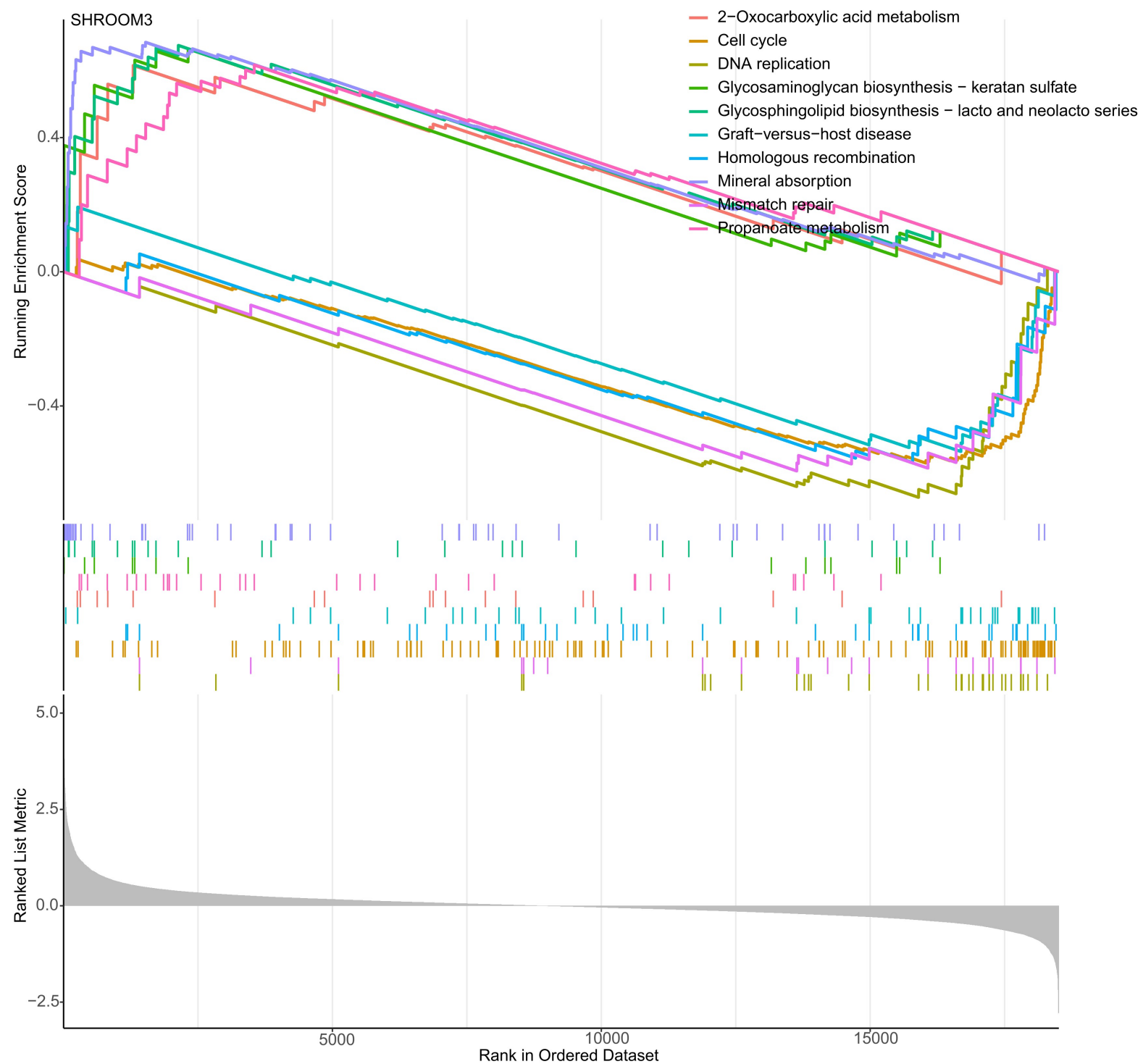

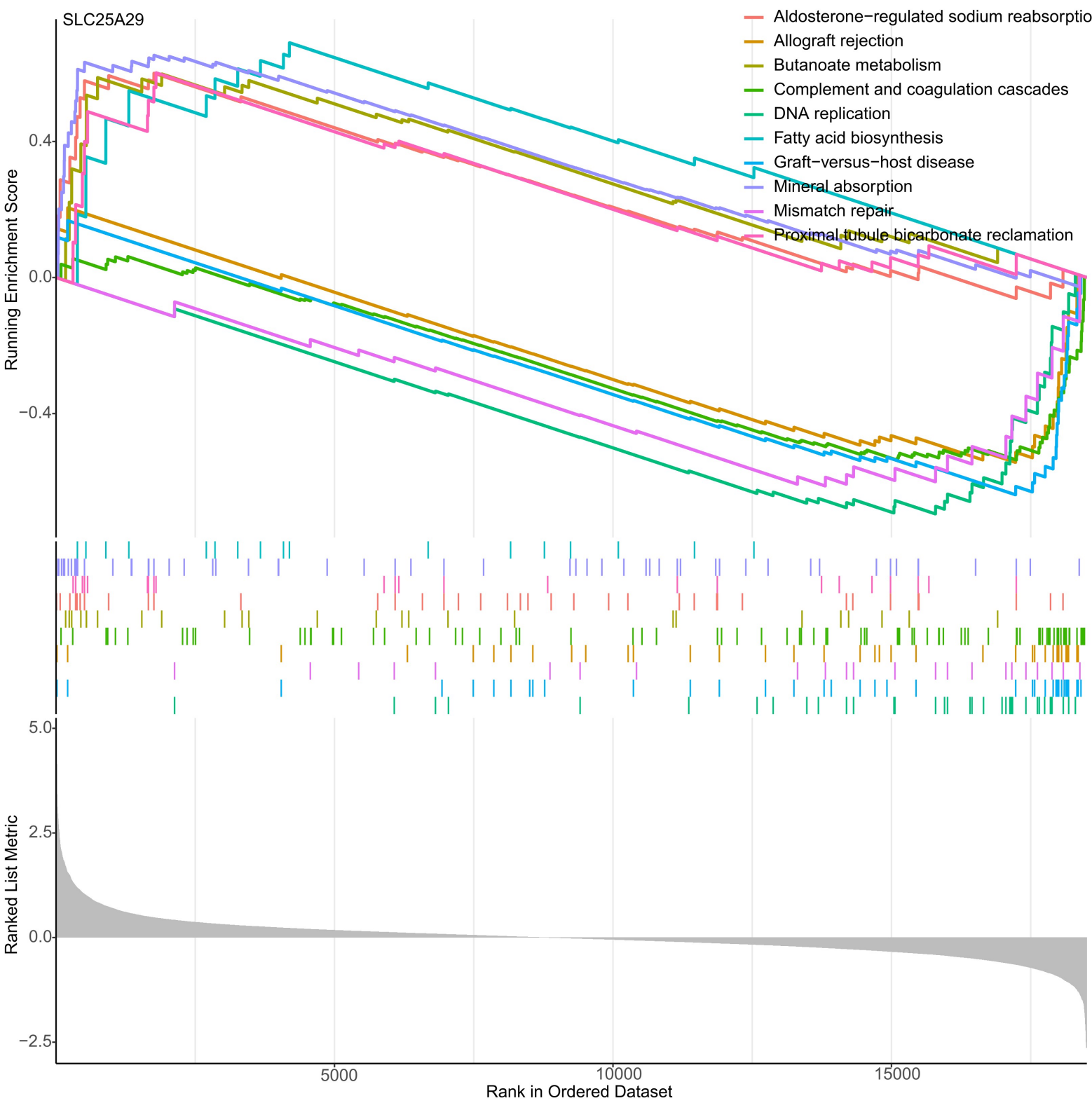

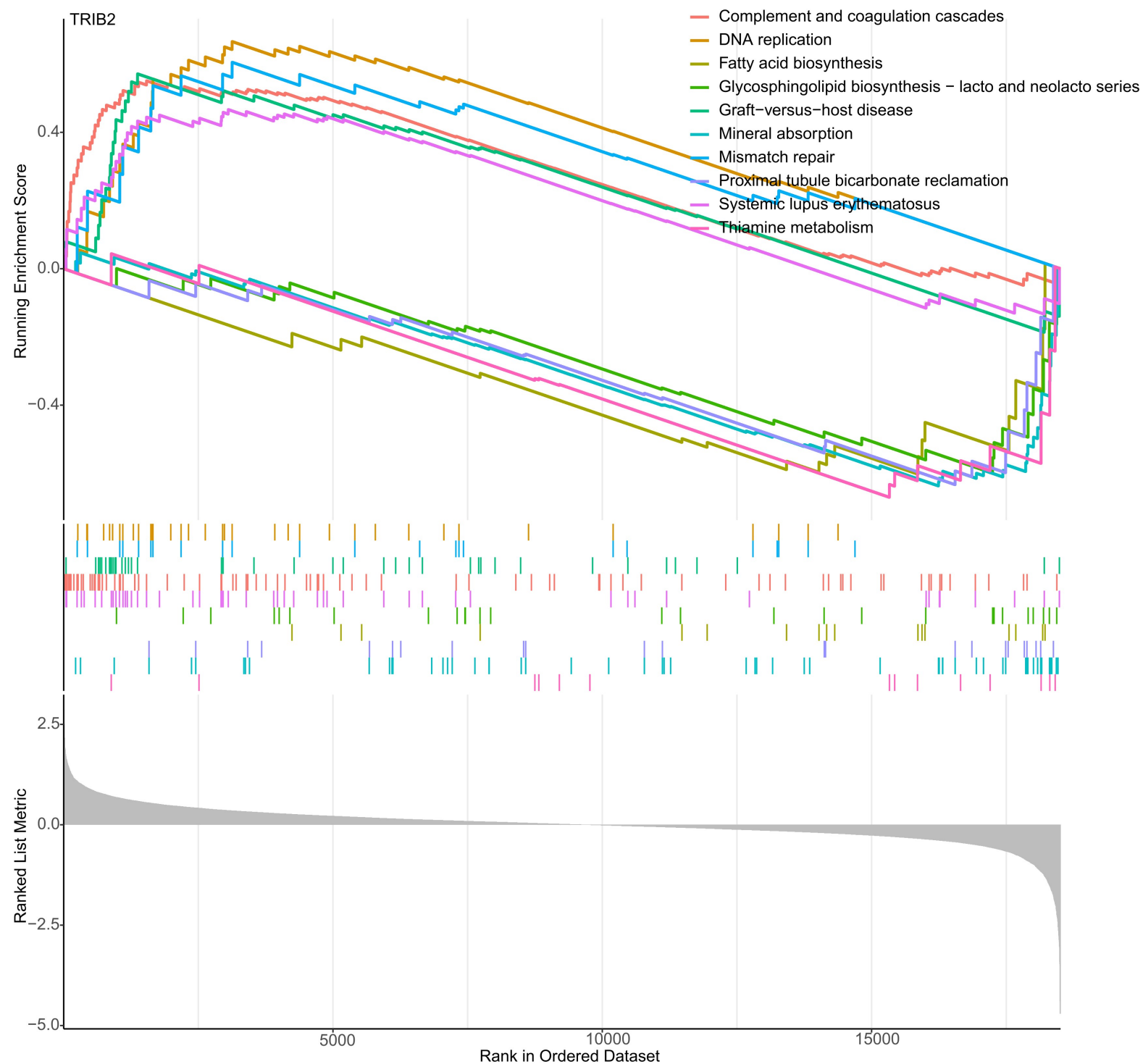

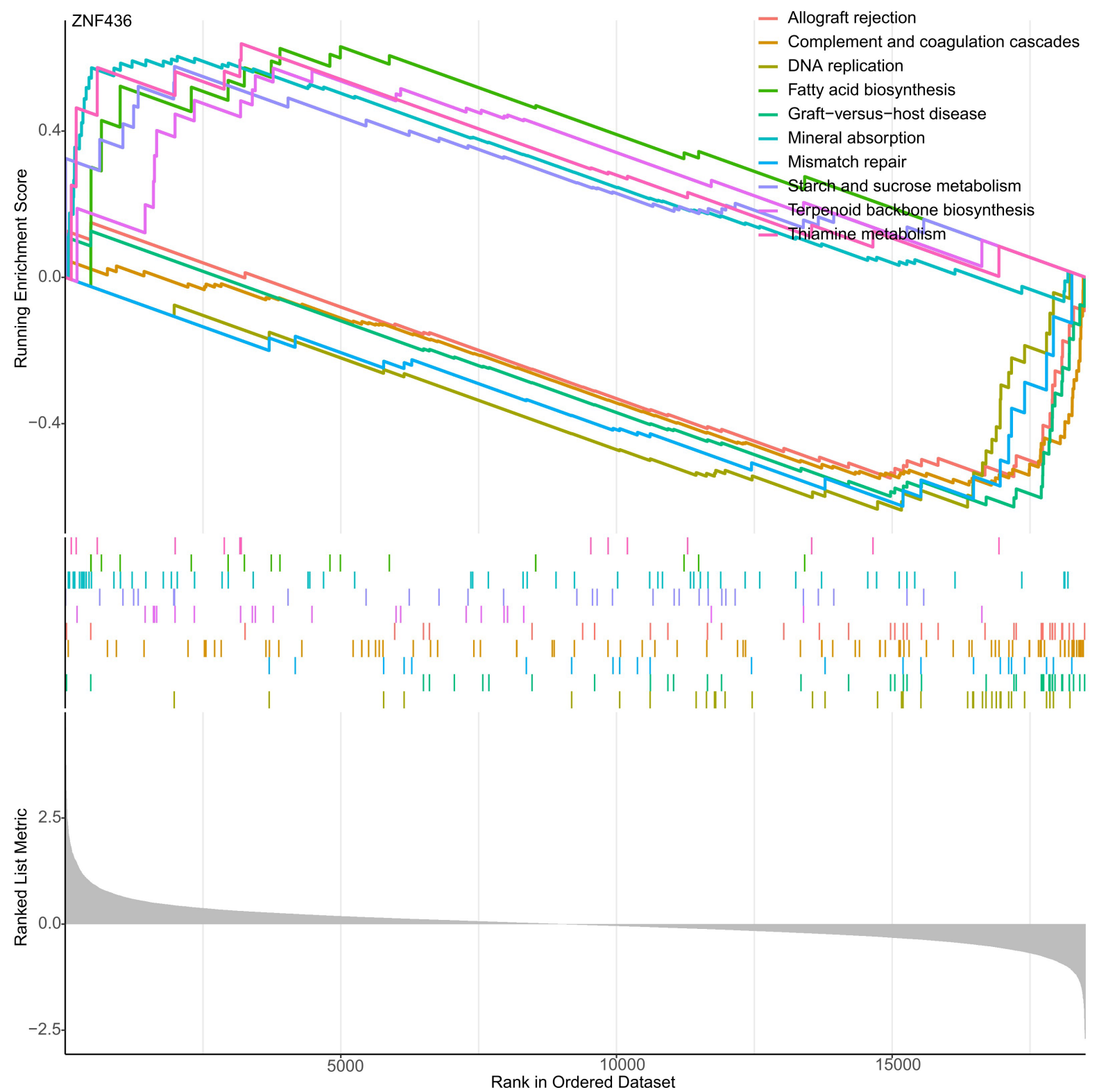

Figure S2. Single-gene GSEA in the GSE111974 dataset.

Supplement: Supplementary file 1 [file biomolecules-13-00406-s001.zip › Supplementary Figure S2.pdf]
