# Supplementary material for: Potential Biomarkers and Endometrial Immune Microenvironment in Recurrent Implantation Failure
Source: Biomolecules. 2023 Feb 21;13(3):406. doi: 10.3390/biom13030406 (PMC10046278; doi:10.3390/biom13030406)
Supplement: Supplementary file 1 [file Supplementary Figure S3.pdf › Supplementary Figure S3.pdf]

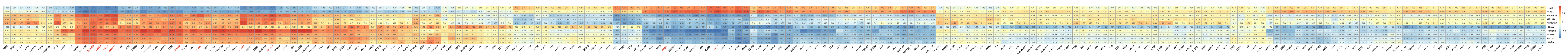

Figure S3. Heatmap of the correlations between ten hub genes and ERA in the GSE111974 dataset.
